# Editorial: Targeting the Tumor Microenvironment for a More Effective and Efficient Cancer Immunotherapy

**DOI:** 10.3389/fimmu.2020.00933

**Published:** 2020-05-15

**Authors:** Salem Chouaib, James Lorens

**Affiliations:** ^1^Department of Experimental Oncology, Thumbay Research Institute for Precision Medicine, Gulf Medical University, Ajman, United Arab Emirates; ^2^INSERM UMR 1186, Gustave Roussy, Université Paris-Saclay, Villejuif, France; ^3^Department of Biomedicine, Centre for Cancer Biomarkers (CCBIO) University of Bergen, Bergen, Norway

**Keywords:** EMT - epithelial to mesenchymal transition, hypoxia, tumor microenvironment, anti-PD 1, T cells

Carcinomas are no longer considered a singular mass of tumor cells; but rather a complex and dynamic pseudo-organ, comprising transformed epithelial cells residing within a complex microenvironment with unique physiology, rich in different non-malignant cell types that interact physically and via paracrine signaling molecules. Immunologists were central to this fundamental re-evaluation of cancer by highlighting the continuous dialogue between immune cells and their cancer targets. Deeper understanding the role of the tumor microenvironment (TME) during cancer initiation and progression is critical both to further cancer biology and as a source of improved molecular diagnostics and therapeutics. The TME is an integral part of tumor physiology that nurtures the malignant process. Many reports indicate that a fundamental albeit deranged relationship between tumor and stromal cells is essential for tumor cell growth, progression, and development of life-threatening metastasis. Therefore, insight into this interaction and the underlying signaling, transcriptional, and metabolic pathways, can reveal valuable opportunities for therapeutic intervention during cancer progression.

The TME is composed of proliferating tumor cells, blood vessels, infiltrating inflammatory cells, and a variety of other associated stromal cells. Dynamic crosstalk between malignant cells and the tumor stroma in the TME determines the trajectory of tumor progression, its aggressiveness, heterogeneity, and response to cancer treatment. How the TME imposes challenges for cancer cells, including physical pressures, oxidative stress, nutrient deprivation, hypoxia, and immune surveillance will be discussed in this special issue.

From an immunological point of view, there is a paradoxical coexistence of tumor and tumor antigen-specific CD8 T cells in cancer patients. Cancer immune resistance arises from multiple negative immunoregulatory pathways that impede T cell-mediated tumor destruction. Tumor stroma components engage in an active and complex molecular cross-talk that compromises immunological recognition of tumors by killer immune cells. This immune suppressive shaping of the TME may be considered an initial immune checkpoint. Although immune checkpoint blockers (anti-PD-1, anti-PD-L1, anti-CTLA-4) on T cells provide improved survival in various metastatic cancer types, a high fraction of cancer patients fail these immunotherapeutic interventions. Strong evidence indicates that neoplastic cell responses to immunotherapy are not solely dependent on the qualitative and/or quantitative features of the T cells or the complexity of the genomic aberrations they harbor, but is also regulated by numerous dynamic properties of the TME. Among the microenvironment factors that play a dominant role in determining therapeutic responses to immunotherapy, hypoxia is central: It is a hallmark of most tumors with the potential for mediating metabolic and phenotypic changes (cell plasticity) as well as direct immune suppression. As a pervasive feature of the TME, hypoxia plays also a significant role in cancer progression and ultimately clinical outcome. One key cellular consequence of hypoxic stress is the dysregulation of DNA repair pathways, which contributes to the genomic instability and mutator phenotype observed in human cancers. Recently, cell plasticity has emerged as potential contributor to therapy evasion through regulation of cancer cell phenotypic and functional heterogeneity. It is now appreciated that microenvironmental stress during tumor development is frequently accompanied by cellular plasticity such as Epithelial-Mesenchymal transition (EMT) that facilitate adaption and selection of lethal cancer clones. Targeting carcinoma cell plasticity is in this regard an important strategy to better control the emergence of resistant variants and ensure a more effective cancer therapy.

With complex mechanisms of resistance limiting the efficacy of checkpoint inhibitor monotherapy, it is critical at present to develop combination approaches to allow more patients to benefit from immunotherapy. In this respect, immunotherapies are more effective when combined with agents that modulate the TME to overcome tumor tolerance and resistance, two key therapeutic challenges. A major barrier remains: How to shape the immunosuppressive TME to promote T cell effector function and overcome tumor immune resistance. Knowing the key suppressive and resistance mechanisms associated with the complexity of the TME will provide the means to break immune tolerance, develop new combinatorial therapeutic strategies and tailor efficient treatments. A better understanding of the crosstalk between signaling pathways and metabolic alterations that drive therapeutic resistance will provide the insight to develop novel therapeutic strategies. The TME is indeed an important target for anti-cancer therapy. Cancer patients will ultimately benefit from drug combinations based on an understanding of the tumor pseudo-organ and not just its individual components.

The aim of this special issue is to provide a comprehensive review of recent understanding of how TME influence on tumor immune resistance and suppression, with a particular focus on current therapeutic strategies to target molecular mechanisms that create and sustain the immune hostile tumor microenvironment. Broadening the clinical applicability of treatments in oncoimmunology requires an improved understanding of the mechanisms, in particular cellular plasticity, complexity, and hostility of the TME, that limit current cancer immunotherapy This reinvigorating of the anti-tumor immune response by targeting the TME can improve cancer treatment.

The papers written by experts in this special issue illustrate how far the field has advanced, but also remind us of the extent of its complexity. This issue offers a current perspective of important aspects of the TME in immune regulation and that impact cancer immunotherapy.

## Author Contributions

All authors listed have made a substantial, direct and intellectual contribution to the work, and approved it for publication.

## Conflict of Interest

The authors declare that the research was conducted in the absence of any commercial or financial relationships that could be construed as a potential conflict of interest.

